# A Change in the Educational Landscape of Regional Anesthesia: A National Survey of Anesthesiology Residency Programs to Assess the Correlation Between the Frequency of Teaching a Block Technique and Clinical Importance

**DOI:** 10.7759/cureus.37869

**Published:** 2023-04-20

**Authors:** Alberto Ardon, Elird Bojaxhi, Steven Clendenen, Robert McClain, Nigel Gillespie, Christopher Robards, Roy Greengrass

**Affiliations:** 1 Anesthesiology, Mayo Clinic, Jacksonville, USA; 2 Anesthesiology and Perioperative Medicine, Mayo Clinic, Jacksonville, USA

**Keywords:** block frequency, education, nerve blocks, importance, regional anesthesia education

## Abstract

Introduction

Variability regarding which blocks are performed most often can be quite high among anesthesiology residency training programs. Which techniques are viewed by residency programs as “critical” for their graduates to know can also be inconsistent. We administered a national survey to investigate correlations between the cited importance of techniques and the relative frequency with which they are being taught.

Materials and methods

A three-round modified Delphi method was used to develop the survey. The final survey was sent to 143 training programs across the United States. The surveys collected information on the frequency with which thoracic epidural blocks, truncal blocks, and peripheral blocks were taught. The respondents were also asked to rate how critical each technique is to learn during residency. A correlation between the relative frequency of block teaching and cited importance to education was calculated using Kendall’s Tau statistic.

Results

Among truncal procedures, transversus abdominis plane (TAP) block and thoracic epidural blocks were frequently viewed as “indispensable for daily practice.” Among peripheral nerve blocks, interscalene, supraclavicular, adductor, and popliteal blocks were frequently viewed as indispensable. All truncal blocks showed a strong correlation between the relative frequency of block teaching and cited importance to education. However, the frequency of teaching interscalene, supraclavicular, femoral, and popliteal blocks failed to correlate with their reported importance ranking.

Conclusions

Perceived importance was significantly associated with the reported frequency of block teaching for all truncal and peripheral blocks except for interscalene, supraclavicular, femoral, and popliteal. The lack of correlation between the frequency of teaching and perceived importance is reflective of a changing educational landscape.

## Introduction

Regional anesthesia has become a highly important aspect of everyday anesthesia practice. One study estimated that over 500,000 peripheral nerve blocks are performed per year for orthopedic surgery alone [1]. Today’s residency graduates are expected, by both surgeons and colleagues, to be well-versed in regional anesthesia techniques and to be able to provide effective and consistent peripheral nerve blockade for patients. The current Accreditation Council for Graduate Medical Education (ACGME) anesthesia residency guidelines require the performance of 40 peripheral nerve blocks during training but do not specify which types of blocks should be included [2]. In 2010, the American Society of Regional Anesthesia and Pain Medicine (ASRA) and the European Society of Regional Anaesthesia and Pain Therapy (ESRA) published educational guidelines that identified tasks, core competencies, curriculum content, and training pathways to be achieved during training [3]. Although advisories such as these provide an effective objective framework and useful rubrics to assess trainee performance, they do not provide guidance regarding which nerve block techniques should be the focus of education. Notably, these guidelines did not identify a recommended number of procedures or frequency of performance to achieve proficiency [4].

Not surprisingly, variability regarding which blocks are performed most often can be quite high among residency training programs. The variability in the utilization of a specific block can be a consequence of a variety of reasons, including practice-related constraints, the number of regional anesthetic techniques available for a surgical intervention, surgeon preferences, and educational biases. Similarly, which techniques are viewed by residency programs as “critical” for their graduates to know, and thus focused upon during training, can be inconsistent in practice and is largely unknown in the literature. To date, no study has examined the frequency and type of regional anesthetic techniques that are currently being taught by training programs across the United States and concurrently examined in a systematic fashion which techniques are considered by programs as “critical” for trainees to master. In this study, we administered a national survey to assess the relative frequencies and relative importance of different block techniques across US anesthesia residency programs and investigate a correlation between the cited importance of a particular technique and the relative frequency with which it is being taught.

## Materials and methods

The study was approved by the Mayo Clinic Institutional Review Board (IRB) (project number: 19-011978); the requirement for written and informed consent was waived by the IRB. Clinical trial registration was not required. A three-round modified Delphi method was used to develop the cross-sectional survey. The Checklist for Reporting of Survey Studies (CROSS) was used to develop and report the survey [5]. Consensus for survey questions was achieved with 75% affirmative votes as described by Diamond et al. [6]. The survey included 47 multiple-choice and Likert scale questions. Once survey consensus was achieved, a pilot survey was sent to the residency directors at the Mayo Clinic campuses in Rochester (Minnesota), Jacksonville (Florida), and Scottsdale (Arizona). A finalized survey was developed based on feedback from these sites utilizing the same modified Delphi method. This final survey was sent to program directors or acute pain service directors at 143 training programs using the Research Electronic Data Capture (REDCap) software (Vanderbilt University, Nashville, TN) in October 2020. Email addresses were obtained from program websites. Survey links were individualized; the researchers were blinded regarding which individuals or programs responded to the surveys. Only one response was allowed per emailed link or program. One completion reminder was sent to all nonrespondents prior to the pre-assigned survey closure date (December 31, 2020). The final survey was not sent to Mayo Clinic sites so as to not introduce home institution bias.

The surveys collected information on the number of thoracic epidural blocks, truncal blocks (erector spinae plane {ESP}, paravertebral, lumbar plexus, transversus abdominis plane {TAP}, quadratus lumborum, rectus sheath, and pectoralis {PECS}), and peripheral blocks (interscalene, supraclavicular, infraclavicular, axillary, femoral, adductor, popliteal, infiltration between popliteal artery and capsule of the knee {iPACK}, and ankle) performed per week. Obstetric epidurals were excluded from the study. For each type of block, the surveys asked educators to rate how frequently the technique was taught during training (1=never/rarely, 2=sometimes, 3=frequently, or 4=always) and how critical the technique is to learn for daily clinical practice (1=not critical at all, 2=somewhat critical, 3=very critical, or 4=indispensable for daily practice). These Likert scales were defined for the participants. Data analysis was performed with AcaStat (AcaStat Software, Winter Garden, FL). After the calculation of descriptive statistics, a correlation between the relative frequency of block teaching and cited importance to education was calculated using Kendall’s Tau statistic. A Tau correlation coefficient above 0.35 was considered a strong relationship.

## Results

Twenty-eight programs responded with a response rate of 19.6%. Most programs were from the East Coast (21.4%) or southeast (25%) regions of the United States. The majority of programs had greater than 30 residents (71.43%), had an established acute pain service (96.43%), and performed more than 40 peripheral nerve blocks per week (64.29%) (Table 1).

**Table 1:** Respondent program characteristics (n=28) RA, regional anesthesia; RAAPM, regional anesthesiology and acute pain medicine

		N (%)
Region	East Coast	6 (21.4%)
	Southeast	7 (25%)
	Midwest	5 (17.8%)
	Southwest	2 (7.1%)
	West Coast	5 (17.8%)
	No response	3 (10.7%)
Number of residents	0-10	2 (7.14%)
	11-20	3 (10.71%)
	20-30	3 (10.71%)
	>30	20 (71.43%)
Number of faculty who teach RA	1-5	4 (14.29%)
	6-10	9 (32.14%)
	11-15	6 (21.14%)
	>15	9 (32.14%)
RAAPM fellowship	Yes	14 (50%)
	No	14 (50%)
Acute pain service	Yes	27 (96.43%)
	No	1 (3.57%)
RA procedures per week: epidurals	0-10	17 (60.71%)
	11-20	6 (21.43%)
	21-40	5 (17.86%)
	>40	0
RA procedures per week: truncal blocks	0-10	5 (17.86%)
	11-20	9 (32.14%)
	21-40	10 (35.71%)
	>40	4 (14.29%)
RA procedures per week: peripheral nerve blocks	0-10	0
	11-20	3 (10.71%)
	21-40	7 (25%)
	>40	18 (64.29%)
Do you believe residents in your program achieve an adequate level of training in regional anesthesia for today’s practice environment?	Yes	25 (89.29%)

**Figure 1:**
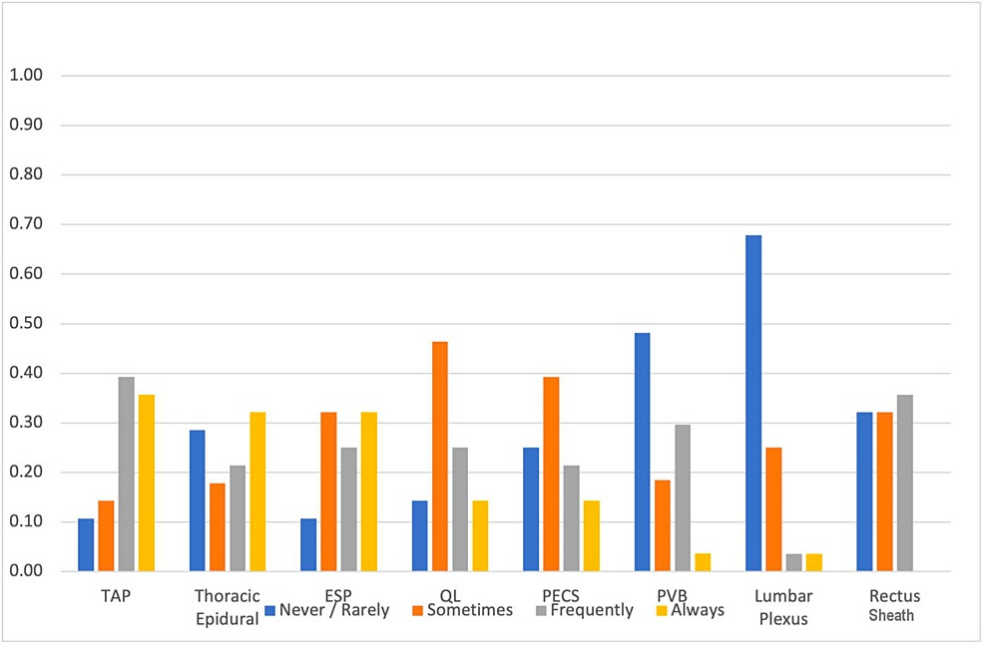
Relative frequencies of teaching of truncal blocks as described by the respondents TAP, transversus abdominis plane; ESP, erector spinae plane; QL, quadratus lumborum; PECS, pectoralis; PVB, paravertebral block

Among truncal procedures, the blocks commonly described as being performed “always” were transversus abdominis plane (TAP) (35.71%), thoracic epidural (32.14%), and erector spinae plane (32.14%) (Figure 1). Paravertebral and lumbar plexus blocks were the least likely to be performed.

**Figure 2:**
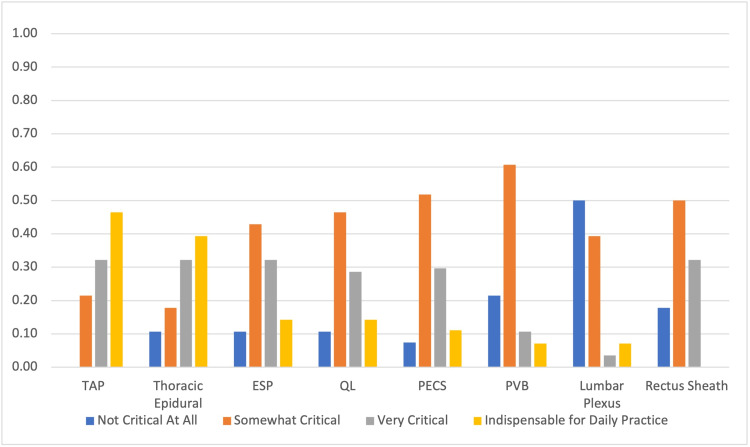
Assessment of the importance of truncal blocks as reported by the respondents TAP, transversus abdominis plane; ESP, erector spinae plane; QL, quadratus lumborum; PECS, pectoralis; PVB, paravertebral block

Concurrently, TAP (46.43%) and thoracic epidurals (39.29%) were more frequently viewed as “indispensable for daily practice” (Figure 2), while lumbar plexus and paravertebral blocks were frequently viewed as “not critical at all for daily practice.”

**Figure 3:**
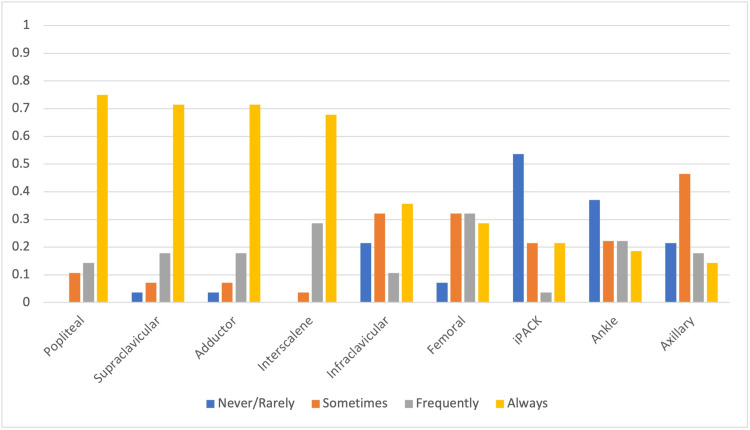
Relative frequencies of teaching of peripheral blocks as described by the respondents iPACK: infiltration between popliteal artery and capsule of the knee

Among peripheral nerve blocks, the blocks commonly described as being performed “always” were popliteal (75%), supraclavicular (71.4%), adductor canal (71.43%), and interscalene (67.86%) (Figure 3), while ankle and iPACK blocks were least likely to be taught.

**Figure 4:**
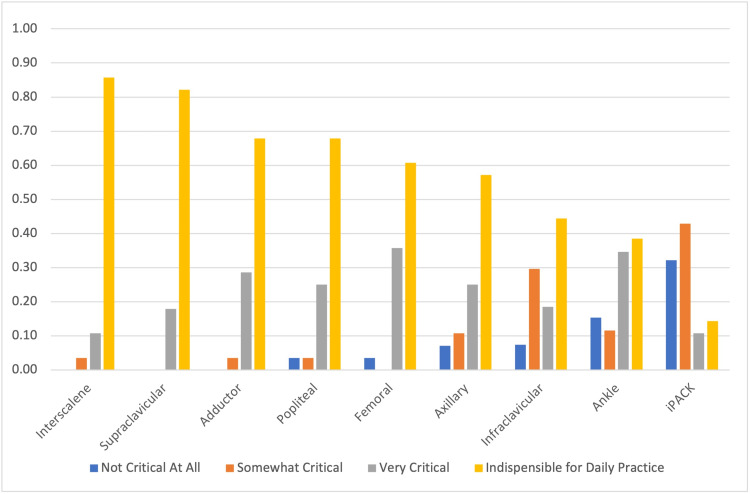
Assessment of the importance of peripheral blocks as reported by the respondents iPACK: infiltration between popliteal artery and capsule of the knee

Interscalene (85.71%), supraclavicular (82.14%), adductor (67.86%), and popliteal (67.86%) blocks were more frequently viewed as “indispensable for daily practice” (Figure 4). Conversely, ankle and iPACK blocks were more frequently viewed as “not critical at all.”

The most significant reported obstacles to teaching regional anesthesia were limited time (53.57%) and limited surgical interest (46.43%) (Table 2).

**Table 2:** Most significant obstacles to teaching regional anesthesia as reported by the respondents RA: regional anesthesia

	N (%)
Limited time	15 (53.57%)
Limited surgical interest	13 (46.43%)
Limited number of procedures amenable to RA	9 (32.14%)
Limited opportunities to learn	6 (21.43%)
Limited resources	3 (10.71%)

In regard to the association between the frequency of teaching and relative importance, all truncal blocks showed a strong correlation between the relative frequency of block teaching and cited importance to education (Table 3).

**Table 3:** Correlation between the frequency of block teaching and cited importance to education ESP, erector spinae plane; QL, quadratus lumborum; TAP, transversus abdominus plane; PECS, pectoralis; PVB, paravertebral block; iPACK, infiltration between popliteal artery and capsule of the knee

	Kendall’s Tau
Truncal	
ESP	0.5774 (p=0.0004)
QL	0.5742 (p=0.0006)
Epidural	0.5685 (p=0.0005)
Lumbar plexus	0.5525 (p=0.0013)
TAP	0.5525 (p=0.0012)
PECS	0.5244 (p=0.0015)
PVB	0.4648 (p=0.005)
Rectus	0.3958 (p=0.0203)
Peripheral	
Ankle	0.6099 (p=0.0002)
iPACK	0.5769 (p=0.0004)
Infraclavicular	0.4873 (p=0.0034)
Adductor	0.3652 (p=0.0491)
Axillary	0.3515 (p=0.0391)
Supraclavicular	0.3154 (p=0.0965)
Femoral	0.3125 (p=0.0792)
Interscalene	0.1202 (p=0.5469)
Popliteal	0.0756 (p=0.7008)

Among peripheral blocks, supraclavicular, femoral, interscalene, and popliteal blocks failed to correlate with their reported importance ranking despite their relatively high frequency of teaching.

## Discussion

In our survey, we found that perceived importance was significantly associated with the reported frequency of block teaching for all truncal and all peripheral blocks except for interscalene, supraclavicular, femoral, and popliteal. This latter finding is surprising considering that these peripheral blocks have historically been a strong component of regional anesthesiology training during residency. For the supraclavicular block findings, while a correlation is suggested, it likely fails to gain statistical significance because of the high propensity of the responders to classify the block as “always taught” and either “very critical” or “indispensable for daily practice” (to the exclusion of other categories), resulting in a skewed nonparametric distribution. Certainly, the supraclavicular approach continues to be a commonly taught and important block for trainees to learn during residency. While the femoral block continues to be considered relatively important for resident education, this block also failed to achieve a statistically significant correlation with the frequency of teaching. This lack of correlation may be reflective of a changing clinical role for the femoral block, as its use has decreased compared to adductor blockade for lower extremity analgesia. For example, a 2013 survey by Moon et al. reported the femoral block to be the most performed block by graduating residents [7]. More than 40% of the respondents in our study identified the femoral block as being performed “never/rarely” or “sometimes,” in contrast to the high frequency of adductor canal block.

Finally, the lack of strong correlation for both popliteal and interscalene blocks could be indicative of the beginning of the shift in opinion or a decrease in the opportunity to teach these blocks. The popliteal block may be taught at a high frequency still, but the perceived clinical importance of this block may be changing, perhaps because of the availability of other more distal nerve block techniques such as peroneal or tibial nerve blocks. However, we did not investigate this possibility. The converse situation may be true for the interscalene block, where the frequency of teaching this block does not correlate with its high importance ranking. This may be suggestive of a decrease in the opportunity to teach this block, perhaps secondary to the use of other analgesic techniques for shoulder surgery such as superior truncal blocks or local infiltration by the surgeon. Indeed, limited surgical interest was the second most reported obstacle (46.63%) to teaching regional anesthesia, and this factor may influence the ability to teach any regional anesthetic technique. There is a national trend toward blocks that do not result in motor blockade or hypotension but result in adequate levels of analgesia as part of a multimodal approach to pain control. Truncal blocks tend to achieve both of these goals and, therefore, are more palatable to surgeons despite their somewhat modest analgesic potency.

Of particular concern to the interpretation of the results of this study is whether anesthesia residents are graduating with the necessary technical skills to provide consistently successful and contemporary regional anesthetics for today’s workforce. Dhanjal and colleagues recently assessed regional anesthesia training among military programs and found that while ACGME requirements were met, most residents were reasonably experienced in only four blocks (interscalene, supraclavicular, femoral, and saphenous) and could not be considered proficient in any of these techniques [8]. Moon et al.’s 2013 survey revealed that only 74% of graduating residents had done more than 60 blocks during training, and the most frequently performed blocks were femoral (59% of graduating residents), followed by interscalene (41%) and popliteal (31%) [7]. A more recent national survey by Neal et al. found that in 2015, residents were likely to graduate with having performed a median of 102 peripheral nerve blocks, an improvement from 45 in 2000 [9]. Among those who reported specific nerve block types, 2015 graduates had performed a median of 22 femoral, 15 interscalene, 13 supraclavicular, 13 popliteal, and 10 saphenous blocks. While the overall increase in peripheral nerve block performance during training from 2000 to 2015 is encouraging, relatively low frequencies of specific techniques could be troubling. If one assumes, as identified by previous studies, that at least 25-40 blocks of any one type are required to achieve proficiency or for trainees to feel comfortable [10], performing a high number of overall blocks during training is paramount to establishing proficiency given the increasing variety of blocks used in practice. This becomes especially critical for techniques that are performed less often across academic centers, such as thoracic epidural catheters, paravertebral blocks, and iPACK blocks, as indicated by our results. Indeed, 60.71% of programs reported performing 10 or less epidural catheters per week, and approximately 30% of programs classified thoracic epidural catheters as being performed never or rarely. As educators, we should be aware of any changes in the educational landscape that may affect learning opportunities for our trainees. Nevertheless, across all blocks, 89.29% of respondents in our study affirmed that graduating residents achieved an adequate level of regional anesthesia training for today’s practice environment.

This study has several limitations. The response rate of 19.6% reflects 28 programs that participated in this survey. This limited number of responses among the 143 anesthesia residency training programs may introduce sampling bias. For example, the majority of survey responses were from programs who had more than 30 residents. Thus, our results may not be reflective of usual practices and resources available at smaller programs. Likewise, cluster sampling may have occurred despite a wide geographic distribution of respondents. Furthermore, our study does not account for newer block techniques that may have become more popular since the administration of the survey. Additionally, this survey does account for the learners’ success in competency-based assessments, which have been shown to be strong prognosticators of skills-based learning [11-13]. Finally, this survey did not sample trainees or practicing anesthesiologists. Assessing resident opinions regarding the breadth and efficacy of their regional anesthesia training would provide an additional layer of context for the interpretation of our results. Similarly, surveying anesthesiologists who have recently graduated from residency would potentially offer practical data regarding the importance of frequently taught blocks and their utility upon entering clinical practice.

## Conclusions

Our study suggests that in today’s training environment, anesthesiology residents are trained in a wide variety of truncal and peripheral nerve block techniques. For truncal blocks, the frequency of block teaching correlates with the perceived importance of teaching that technique. However, among peripheral nerve blocks, this relationship did not exist for supraclavicular, femoral, interscalene, and popliteal blocks. The lack of a strong correlation between teaching and perceived clinical importance is reflective of the changing educational landscape for peripheral nerve blocks.
